# Supplementary material for: SyNDI: synchronous network data integration framework
Source: BMC Bioinformatics. 2018 Nov 6;19:403. doi: 10.1186/s12859-018-2426-5 (PMC6219086; doi:10.1186/s12859-018-2426-5)
Supplement: Supplementary file 5 — Files of the example presented in Additional file 4. This additional file is a zip package that contains all files used in Additional file 4. Detailed descriptions of each of these files are presented in a README file included in this zip package. (GZ 30411 kb) [file 12859_2018_2426_MOESM5_ESM.gz › SyNDITools/Meme2Fimo/meme_4.9.0_4.tar.gz!/meme_4.9.0/website/html/meme-download.html]

MEME - Submission form


|  |  |
| --- | --- |
|  | MEME Suite The MEME Suite web application requires the use of JavaScript   Javascript doesn't seem to be available on your browser.  Download Software  The MEME Suite software is available for FREE interactive use via the web.  Alternatively, you can download the software for installation and **non-profit use** on your own computer. Please read the Copyright for terms and conditions before downloading the software. **For-profit** licenses are also available; click here for details.  The downloadable software includes the complete source code for the MEME suite, and instructions on how to install and test them. The online installation guide is Installation instructions.  When you install the MEME Suite software on your own computer, you can use many features not available with the interactive versions. Refer to the commmand-line documentation for information on these features.   - Download the MEME Suite source code and databases - Installation Guide - Copyright - License [PDF] (for-profit only) - View command-line documentation |
